# CRP/albumin ratio and WBC values correlate with Ki-67 and survival in glioblastoma multiforme

**DOI:** 10.3389/fonc.2025.1612212

**Published:** 2025-06-26

**Authors:** Güven Gürsoy

**Affiliations:** Department of Neurosurgery, Faculty of Medicine, Muğla Sıtkı Koçman University, Muğla, Türkiye

**Keywords:** glioblastoma multiforme, prognostic factor, inflammatory marker, overall survival, glioma

## Abstract

**Introduction:**

Glioblastoma multiforme (GBM) is an aggressive central nervous system tumor that results in poor overall survival due to its rapid and aggressive course. Prognostic indicators are important in treatment strategies. This study aimed to investigate the effectiveness of inflammatory markers C-reactive protein (CRP)/albumin ratio, and white blood cell (WBC) count, as well as the pathological indicator Ki-67, for survival prediction and prognosis and their superiority.

**Material and methods:**

The demographic data of the patients, Glasgow Coma Scale (GCS), WBC, CRP, albumin, CRP/albumin values, Ki-67 values ​​at pathological diagnosis, and overall survival were examined in this study. Adults over the age of 18 who underwent gross total surgery in a single center and whose pathological diagnosis was glioblastoma multiforme, who received radiotherapy and chemotherapy after surgery, were included. Patients with chronic and comorbid diseases were excluded because of their potential to affect the parameters to be examined.

**Results:**

Among the 133 GBM cases, between 23 and 72 years of age were included, and 72.9% (n: 97) had a survival less than one year survival. Ki-67, WBC, CRP, albumin, and the CRP/albumin ratios were found to be statistically significant for 1-year overall survival (p values ​​in order: <0.001, <0.001, <0.001, 0.013, <0.001). The receiver operating characteristic (ROC) curve analysis revealed significant cutoff value at 22.5 for Ki-67, 0.70 for CRP/albumin ratio, and 7.42 for the WBC count for 1-year survival.

**Conclusion:**

The WBC count, CRP/albumin ratio, and Ki-67 parameters can be used to predict 1-year overall survival in patients with glioblastoma multiforme. These findings emphasize the need for more prognostic scoring models and evaluations of the role of inflammation in GBM prognosis.

## Introduction

Glioblastoma multiforme (GBM) is one of the most aggressive malignancies of the whole body and the most common malignant primary tumor of the central nervous system (CNS) (1). GBM accounts for approximately half of malignant CNS tumors with a 5-year survival of 7.2% (2). Because of the complex interaction of tumor cells within the brain environment and extracellular matrix components (3), maximal safe resection followed by postoperative temozolomide (TMZ) concurrent with or adjuvant to partial brain radiotherapy (RT) is the highest-quality level treatment for GBM patients (4).

Prognostic factors in GBM patients are age, Karnofsky performance status (KPS), neurologic function status, extent of surgery, the methylation status of O6-methylguanine-DNA methyltransferase (MGMT), isocitrate dehydrogenase-1 (IDH-1) and IDH-2 status, and administration of EORTCH-NCIC protocol (5, 6). Preoperative white blood cell (WBC) and C-reactive protein (CRP) values (7) and C-reactive protein to Albumin ratio (CRP/Albumin) in patients treated with radiotherapy and concurrent plus adjuvant temozolomide (8) are also prognostic factors.

Ki-67 is a nonhistone nuclear protein that is expressed during the proliferative cell cycle. It is associated with the transcription of ribosomal ribonucleic acid (rRNA). Ki-67 is expressed throughout all active cell cycle phases except in the resting cell phase, G0. Ki-67 expression is high in malignant cells (9), but the prognostic value of Ki-67 for GBM is controversial (10, 11).

Prognostic comparisons between simple biochemical markers and pathological findings of tissues obtained via surgical procedures have not been widely studied to our knowledge. In this study, it was aimed to compare biochemical inflammatory markers with pathological indicators in terms of survival prediction and prognosis in patients with glioblastoma multiforme at a single institution.

## Materials and methods

This retrospective clinical study was designed to investigate the relationships among the CRP/albumin ratio, WBC count, and the proliferation marker Ki-67, for the overall survival prediction in pathologically diagnosed glioblastoma multiforme patients, who were operated on at Muğla Training and Research Hospital (affiliated with Muğla Sıtkı Koçman University) by the same surgeon between March 2017 and March 2024.

The study included both male and female patients aged over 18 years without any comorbidities. All patients underwent gross total surgery and were pathologically diagnosed with glioblastoma multiforme. Chemotherapy and radiotherapy were added to all patients during the postoperative period. Median number of radiotherapy treatment time was 3 weeks and median number of dose was 40 Gy with 15 fractions. The only regime for chemotherapy was temozolomide. Patients under 18 years of age, patients with comorbidities due to the potential to affect laboratory parameters, patients with Karnofsky performance status (KPS) scores below 70, patients who received radiotherapy and/or chemotherapy prior to surgery, patients who underwent surgery more than once, and patients whose data were not available were excluded from the study. Subtotal resections or biopsy procedures during surgery were also excluded. The anatomical location of the lesion was not considered an inclusion or exclusion criterion for the study.

Age and sex information, Glasgow Coma Scale (GCS), preoperative WBC, CRP, albumin values, and CRP/albumin ratio, Ki-67 values ​​in the pathology report, and postoperative survival were documented via archive scanning.

### Statistical analysis

Statistical analyses were performed via Statistical Package for the Social Sciences (SPSS) software version 30.0. The variables were investigated using visual (histograms, probability plots) and analytic methods (Kolmogorov-Smirnov/Shapiro-Wilk’s test) to determine whether they were normally distributed. Descriptive analyses are presented as medians for the nonnormally distributed and ordinal variables. The Kruskal-Wallis tests were conducted to compare non-normally distributed parameters. The Mann-Whitney U test was performed to test the significance of pairwise differences using Bonferroni correction to adjust for multiple comparisons.

The possible factors identified with univariate analyses were further entered into the logistic regression analysis to determine independent predictors of patient survey results for the multivariate analysis.

The correlation coefficients and their significance were calculated using the Spearman test while investigating the associations between nonnormally distributed and/or ordinal variables.

The capacity of Ki-67, WBC, and the CRP/albumin ratio values in predicting the presence of patients survey was analyzed by using Receiver Operating Characteristics (ROC) curve analysis.

An overall 5% type-I error level was used to infer statistical significance.

Post power levels for Ki-67 and CRP/Albumin ratio were calculated by referencing **Table 1** statistical results. The area under curve (AUC) value of Ki-67 is 0.928 ± 0.019 and the AUC value of CRP/Albumin ratio is 1.872 ± 0.045. The standard AUC value to be tested is 0.5 and the power level calculated for n=133 is 99.4% for Ki-67 and 99.99% for CRP/Albumin ratio.

**Table 1:** Diagnostic scanning and ROC curves for the Ki-67, CRP/Albumin ratio and WBC compared to 1-year overall survey.

Significance of Ki-67 ROC Curve		
Area under the ROC curve (AUROC)	0.928	P-value=0.000
Standard Error	0.021	
95% confidence interval	0.886-0.969	
Cut-off	22.5	
Sensitivity	78.4%	
Specificity	100%	
Significance of CRP/Albumin Ratio ROC Curve		
Area under the ROC curve (AUROC)	0.872
Standard Error	0.044
95% confidence interval	0.785-0.959
Cut-off	0.70
Sensitivity	86.6%
Specificity	83.3%
Significance of WBC ROC Curve	
Area under the ROC curve (AUROC)	0.783
Standard Error	0.040
95% confidence interval	0.706-0.861
Cut-off	7.42
Sensitivity	72.2%
Specificity	72.2%

## Results

A total of 133 patients were included in the study after the exclusion criteria were applied. It was determined that 49.6% (n:66) of the patients were female while 50.4% (n:67) were male, and the average age of all patients was 52.55 ± 11.28 years. The average survival time was 10.33 ± 4.74 months, and 72.9% (n:97) of the patients survived for one year or less.

Ki-67 (p<0.001), WBC (p<0.001), CRP (p<0.001), albumin (p:0.013), and CRP/albumin ratios (p<0.001) were found to be statistically significant when the patients were grouped according to their 1-year survey results (**Table 2**).

**Table 2:** Descriptive data grouped according to 1-year overall survey.

Survey and Variables	Under 1 year (n:97)	Over 1 year (n:36)	Total (n:133)	P-value
Age				
Median	53	55	54	0.429
Maximum	72	70	72	
Minimum	23	29	23	
Gender				
Woman % (n)	51.5% (50)	44.4% (16)	49.6% (66)	0.559
GCS				
Median	15	15	15	0.043
Maximum	15	15	15	
Minimum	11	14	11	
Ki-67, %				
Median	25	17.5	25	<0.001
Maximum	45	20	45	
Minimum	15	10	10	
CRP, mg/L				
Median	5.29	1.73	4.42	<0.001
Maximum	36.91	11.78	36.91	
Minimum	0.6	0.39	0.39	
Albumin, gr/dL				
Median	4.33	4.58	4.43	0.018
Maximum	4.97	4.95	4.97	
Minimum	2.88	3.53	2.88	
WBC, x1000/microL				
Median	9.12	6.7	8.19	<0.001
Maximum	16.47	9.42	16,47	
Minimum	4.25	3.53	3.53	
CRP/Albumin Ratio				
Median	1.27	0.38	0.97	<0.001
Maximum	9.59	3.16	9.59	
Minimum	0.21	0.1	0.1	

Independent factors predicting patient survival were examined using logistic regression analysis in multivariate analysis. These predictors were found to be statistically significant as Ki-67 <0.001, CRP: 0.004, Albumin: 0.022, CRP/Albumin ratio: 0.005. A 1-unit change in Ki-67 decreased the survey by 0.55, CRP level by 0.08, albumin level by 1.35, and CRP/albumin ratio by 1.82 times (**Table 3**). WBC count (p:0.763), GCS score (p:0.526), and patient age (p:0.079) were not statistically significant.

**Table 3:** Logistic regression analysis of factors affecting the 1-year overall survey.

Risk Factor	OR (95% CI)	P-value
Ki-67	0.528 (0.434-0.717)	<0.001
CRP	0.088 (0.016-0.469)	0.004
Albumin	1.353 (1.044-1.754)	0.022
CRP/Albumin Ratio	1.824 (1.257-4.816)	0.005

The correlation matrix revealed a low moderate positive correlation between the patients’ survey and GCS score (r: 0.389), a negative excellent correlation between Ki-67 (r: -0.878), a negative moderate correlation between WBC (r: -0.598), a negative excellent correlation between CRP (r: -0.766), a positive low and insignificant correlation with albumin (r: 0.183), and a negative excellent correlation with the CRP/albumin ratio (r: -0.757). All the predictive factors were found to be statistically highly significant except for the serum albumin concentration (p values ​​<0.001).

When Ki-67 and other factors were examined, there was a low-moderate (r: -0.356) but very significant (p<0.001) negative correlation with the GCS score, a moderate (r: 0.529) and very significant (p<0.001) positive correlation with the WBC count, and a good (in order of r: 0.700, r: 0.683) and very significant correlation with the CRP level and the CRP/albumin ratio (p<0.001). No statistically significant effect was found when evaluating the combined impact of age and Ki-67 levels on survival time and overall survival (p>0.05).

**Figure 1:**
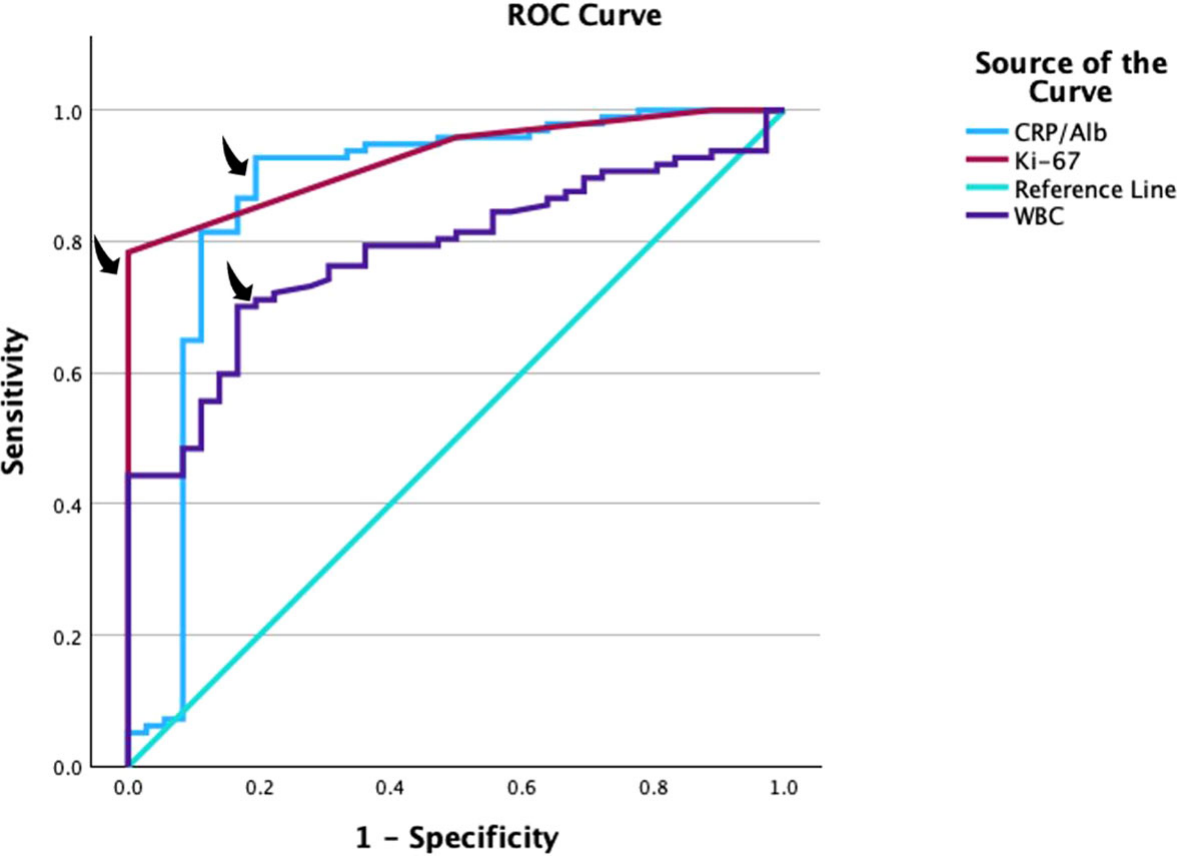
Receiver-operating characteristics (ROC) curves displaying predictive value of CRP/Albumin ratio, Ki-67 and WBC for 1-year overall survey. Arrows display sensitivity/specificity values.

The performance of the Ki-67 index, CRP/albumin ratio, and WBC count in determining patient survival was evaluated via receiver operating characteristic (ROC) analysis. There was a statistically significant difference in the Ki-67 index, CRP/albumin ratio, and WBC count according to prognosis (p=0.00). Area under the curve was 0.928 (CI 95% 0.886-0.969) with 0.021 standard error for Ki-67 and 0.872 (CI 95% 0.785-0.959) with 0.044 standard error for CRP/Albumin ratio, and 0.783 (CI 95% 0.706-0.861) with 0.040 standard error for WBC. The Ki-67 showed significantly higher AUROCs compared to the CRP/Albumin ratio. Results for Ki-67 score were the cut-off point of 22.5, results for CRP/Albumin ratio were the cut-off point of 0.70, and results for WBC score were the cut-off point of 7.42 (**Figure 1**, **Table 1**).

## Discussion

The results of this study revealed that lower Ki-67, CRP, CRP/Albumin ratio, and WBC values are associated with longer lifespan. The relationship between the CRP/Albumin ratio and WBC to Ki-67 index is found to be significant. This suggests that the prognosis estimation provided by biochemical markers and Ki-67 values is similar. More specifically, patients with a Ki-67 index above 22.5, a CRP/Albumin ratio above 0.70, and a WBC value above 7.42 (x1000/microL) have a lower chance of 1-year overall survival after diagnosis.

Inflammatory conditions are known for cancer development in various tissues. Several systemic inflammation markers, such as lymphocyte count and serum albumin, have been identified in various cancer types (12, 13). The definite mechanism of how the CRP/Alb affects the clinical outcomes of the GBM patients has not been explained yet. Local and systemic chronic inflammation assuredly plays pivotal roles in the initiation of gliomagenesis and its malignant progression (14, 15). A multicenter cohort study detected high sensitivity and specificity of preoperative inflammatory markers for glioma diagnosis and differential diagnosis of low-grade glioma from GBM (16). In another study for inflammatory markers in GBM, Pierscianek et al. stated that preoperative WBC and CRP values were confirmed as independent predictors of GBM outcome, with WBC>12/nl having a robust significant association with 1-year and 2-year survival (7). Also, high neutrophil-to-lymphocyte, platelet-to-lymphocyte, and monocyte-to-lymphocyte ratios were found to be independent prognostic factors for overall survival in patients with GBM (17). A meta-analysis in 2,275 patients found a similar association with neutrophil-to-lymphocyte ratio (18). Our study estimated similar results to these studies. The WBC values ​​of patients with overall survival under one year were found to be 4.25-16.47, and those with overall survival over one year were found to be 3.53-9.42. In more precise terms, we found that WBC values ​​over 7.42 (x1000/microL) were significant in predicting the prognosis of overall survival under one year.

Hypoxia and necrotic tumor cell-induced mediators are thought to be the reason for systemic inflammation, causing elevated CRP and reduced albumin levels in any cancer type, including Glioblastoma Multiforme. Since the anabolism of CRP is increased in any particular inflammatory situation that contrasts with the provoked catabolism of Albumin, the CRP and Albumin levels are unquestionably recognized to be strongly and inversely correlated (19). The reactionary secretion of tumor necrosis factor-alpha (TNF-α) and interleukin-6 (IL-6) results in decreased levels of the serum Albumin because of its upregulated catabolism and downregulated hepatic synthesis in similar conditions (20). van den Beld et al. reported that low serum Albumin levels were closely related to high levels of insulin-like growth factor-binding protein 2 (IGFBP-2) and IL-6: other two independent poor prognosticators of GBM (21, 22). Although it may not address the exact mechanism underlying the complicated interplays between the serum levels of CRP, Albumin, IL-6, and IGFBP-2, these results infer that the GBM-provoked local and systemic inflammation plays essential roles in the faith of treatment response. Based on these basic ideas, the ratio of CRP to albumin has been examined in various inflammatory conditions. In a study conducted on 153 GBM patients receiving concurrent radiotherapy and temozolomide treatment, it was reported that median overall survival was significantly reduced with a CRP/Albumin ratio of >0.75 compared to a CRP/Albumin ratio of <0.75 (8). The CRP/Albumin ratio of our patients was observed to be between 0.21 and 9.59 in patients with survival of less than one year, and between 0.1 and 3.16 in patients with survival of more than one year. A CRP/Albumin ratio of more than 0.70 has achieved significant results in predicting the prognosis of survival of less than one year.

Ki-67 has been emphasized as one of the prognostic markers for a long time in Glioblastoma Multiforme. This is a nuclear protein associated with the proliferation phase of a physiological cell cycle, which is specific to the normal or the tumor cell expressing this protein (23). The Ki67 protein has a half-life of only ~1 to 1.5 hour. It is present during all active phases of the cell cycle (G1, S, G2 and M), but is absent in resting cells (G0). In later phases of mitosis (during anaphase and telophase), a sharp decrease in Ki67 levels occurs (24). Expression of the Ki67 protein (pKi67) is associated with the proliferative activity of intrinsic cell populations in malignant tumors. Ki67 protein expression coincides with the transit of cells through mitosis and undergoes phosphorylation and dephosphorylation during mitosis *in vivo*, rendering it susceptible to protease degradation (25). 35% of the Glioblastoma cases were found to have Ki-67 greater than 20% (26). Although the general consensus is that Ki-67 has a role in determining prognosis, different results are found in the literature (27). A Ki-67 proliferation index higher than 22% is found to be predictive of poorer survival in glioblastomas (23). On the contrary, Wong et al. demonstrated that patients with radiotherapy and chemotherapy have a longer survival associated with a high Ki-67 index (28). The Ki-67 index of patients with less than one year overall survival was found to be between 15 and 45, while in those with more than one year it was found to be between 10 and 20. A Ki-67 above 22.5 was found to be a limit value in estimating the 1-year overall survival prognosis in this study.

When biochemical parameters were compared with the Ki-67 pathological index and it was examined which one gave more accurate results in predicting prognosis among them; WBC value, CRP/Albumin ratio, and Ki-67 index gave significant results separately and alone, and it was seen that Ki-67, CRP/Albumin ratio, and WBC values ​​in order were superior to each other.

## Conclusion

Glioblastoma Multiforme is a challenging pathology due to its aggressive course and the low survival time seen in patients. Prognostic indicators have always been important in planning the treatment to be performed and informing patients. WBC and CRP/Albumin ratio indicators obtained with a more noninvasive method compared to surgical techniques can be used for overall survival prediction, as can the Ki-67 index obtained with a surgical procedure. We believe that a new comprehensive scoring model for determining prognosis in Glioblastoma Multiforme patients could be planned, using parameters such as the WBC and CRP/Albumin ratio, which are relatively more accessible and yield similar efficacy results, along with markers like Ki-67 when necessary.

### Limitations

The retrospective design of the database is the major limitation. Although patients with additional diseases were not included in the study due to their potential effects on inflammatory biomarkers, it should not be ignored that conditions such as smoking, history of concomitant epilepsy, and steroid use may affect inflammatory markers. Further prospective studies that can be conducted by correlating pathologically different subtypes of Glioblastoma Multiforme and mutation analyses may reveal more specific results.

## Data Availability

The original contributions presented in the study are included in the article/supplementary material. Further inquiries can be directed to the corresponding author.
